# A novel conformable embolic for selective transarterial embolization of acute hemorrhages: a technical note

**DOI:** 10.1186/s42155-024-00492-0

**Published:** 2024-11-06

**Authors:** Qian Yu, Osman Ahmed, Jiaqi Chen, Yousuf Islam, Brian Funaki, Mikin Patel

**Affiliations:** 1 Department of Radiology, University of Chicago Medical Center, University of Chicago, Chicago, IL 60637 USA; 2 Chicago Medical School, 3333 N Green Bay Rd, North Chicago, IL 60064 USA

## Abstract

**Background:**

Obsidio conformable embolic (OCE, Boston Scientific, MA) is a novel, radiopaque and conformable embolic. The purpose of this report is to describe its use for treatment of acute intra-abdominal hemorrhages.

**Methods and results:**

Three patients presented with acute hemorrhage and were treated with OCE, including post-paracentesis hemorrhage, penetrating trauma to the liver, and blunt trauma in the spleen. All cases were performed under moderate sedation, with hemostasis achieved by end of procedure using less than 1 vial of OCE (0.2-0.4 ml). No severe adverse events occurred. None required repeated treatment.

**Short conclusion:**

OCE is a safe and effective embolic agent for treatment of intra-abdominal or visceral hemorrhage. Future studies with larger sample sizes and longer follow-up are warranted.

##  Background

Existing embolic agents for transarterial embolization (TAE) suffer from potential drawbacks including dependence on an intact coagulation cascade, limited (i.e. temporary) durability, limited real-time visualization under fluoroscopy (i.e. gel foam and particles), catheter adherence, and prolonged preparation time [1, 2]. Obsidio Conformable Embolic (OCE, Boston Scientific, MA) is a radiopaque, conformable, and premixed embolic consisting of porcine gelatin, layered silicate, and tantalum [3, 4]. As a non-Newtonian fluid, it behaves like fluid under high pressure during injection via a microcatheter, whereas it displays properties of a solid material once delivered and fully occludes the lumen endovascularly [4–6]. In theory, the occlusive property of OCE allows it to function independently of coagulation status, with the additional benefit that it does not adhere to the microcatheter like N-butyl cyanoacrylate (NBCA) glue embolic. It is licensed for use in peripheral arteries that are smaller than 3 mm. As a newer embolic, only limited clinical experience has been described, and its property as a non-Newtonian fluid is associated with unique tactile feedback during delivery that is different from existing embolization agents, requiring a learning curve given its material makeup. This technical note describes its use for treating acute intra-abdominal hemorrhage, focusing on its safety, technique, and effectiveness.

##  Main text

###  Technique

**Fig. 1:**
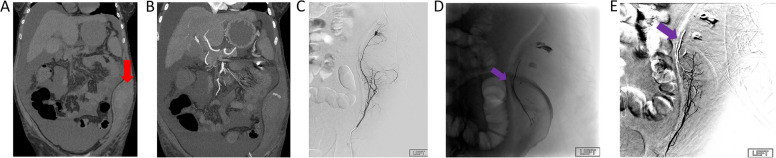
**A** and **B** pre-procedural computed tomography angiogram demonstrated left anterolateral abdominal wall hematoma and contrast extravasation (**A** non-contrast scan, coronal view; **B** arterial phase maximum intensity projection, coronal view; red arrow: hematoma and active bleeding). **C** selective catheter angiogram of the left deep circumflex iliac artery demonstrated contrast extravasation into the lateral abdominal wall soft tissues (red arrow: active bleeding). **D** Long segment arteries were occluded by radiopaque embolic (purple arrow: embolic). **E** Postembolization DSA demonstrated arrest of previously observed extravasation (purple arrow: embolic)

The embolic agent is supplied in a 1 mL sterile syringe and is stored at 2–8 °C fridge with one-year shelf life (Fig. 1). It can be removed and utilized immediately without any prior preparation. The syringe is designed for connection to the back of a microcatheter for direct injection. No pre-loading of solvent (e.g. dimethyl sulfoxide) is required. The embolic can be injected and deployed into the target vessel in a similar fashion to NBCA or ethylene vinyl alcohol (EVOH) based embolic, although with a higher tactile resistance during syringe injection and different, variable viscosity profile as described below. Following the injection, the microcatheter can be removed carefully by withdrawing without risk of catheter entrapment. While not in the device instructions for use, the authors’ experience has allowed for the microcatheter to be thoroughly flushed with saline on the back table and reused.

###  Case 1

A 60-year-old female with decompensated alcoholic cirrhosis, esophageal varices, and portal hypertension underwent bedside paracentesis and developed acute blood loss anemia (Fig. 1). Computed tomography (CT) demonstrated a left abdominal wall hematoma measuring approximately 10.3 × 6.3 cm with associated active extravasation. A digital subtraction angiogram (DSA) demonstrated contrast extravasation of the left deep circumflex iliac artery. A total of 0.4 ml out of 1.0 ml vial OCE was injected with a controlled manner via a 4 Fr Kumpe catheter, occluding a long segment of arteries measuring approximately 7.7 cm. Immediate cessation of flow was achieved on the initial post-embolization angiogram. No procedural-related major complication occurred [7]. Patient was followed for up-to 6-month post embolization without re-bleeding.

### Case 2

**Fig. 2:**
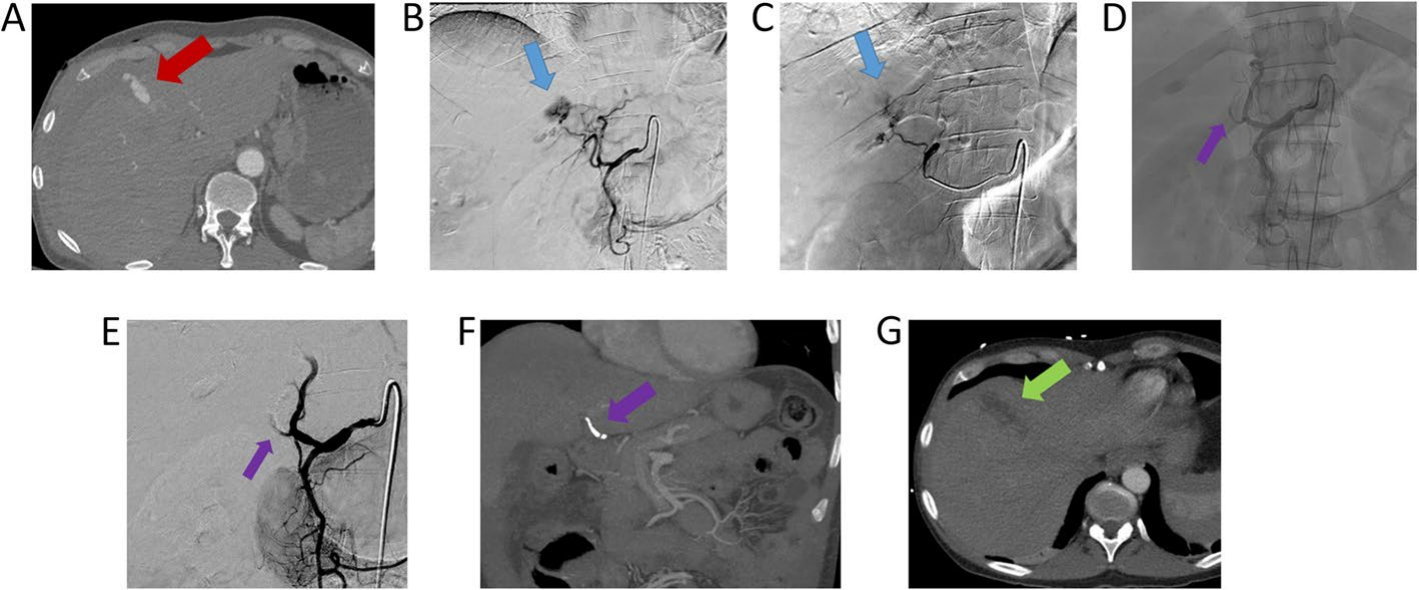
**A** Computed tomography angiogram demonstrated segment 4 contrast extravasation (red arrow). **B** and **C** Celiac and proper hepatic artery angiograms confirmed contrast blush from the segment 4 hepatic artery (blue arrow). **D** and **E** Postembolization angiogram showed resolution of segment 4 blush. Purple arrow: Obsidio embolic (**D** unsubstracted image; **E** subtracted angiogram). **F** and **G** Follow-up computed tomography demonstrated absence of active hemorrhage within the embolization bed (green arrow). Purple Arrow: Obsidio embolic

A 46-year-old male presented with a stab wound to the right upper quadrant (Fig. 2). Computed tomography angiogram (CTA) of the abdomen revealed contrast extravasation from segment IV, which was confirmed with angiography. Superselective embolization of segment 4 A branch was performed using 0.2 ml OCE through a 2.8 Fr microcatheter (Terumo, Europe NV). Post-embolization angiography demonstrated successful cessation of bleeding with no evidence of residual extravasation. No procedural-related major complication occurred [7].

### Case 3

**Fig. 3:**
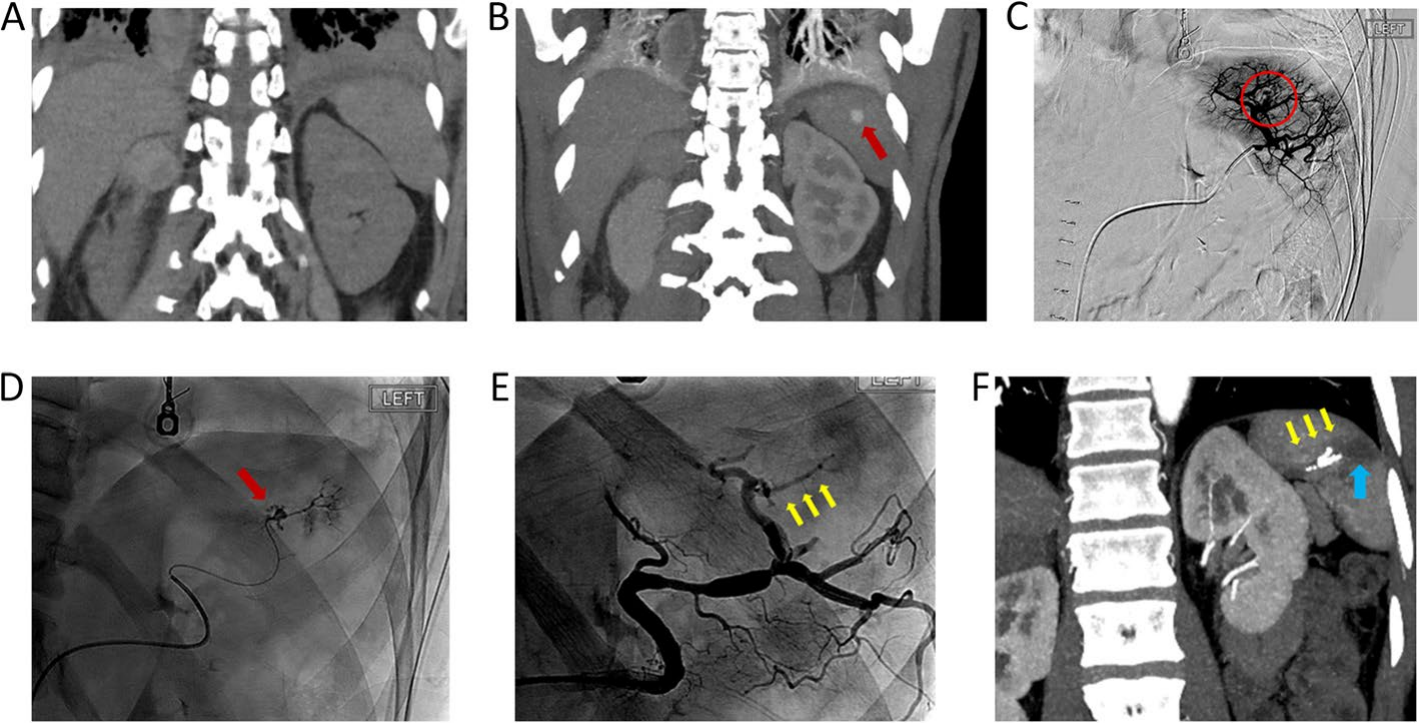
**A** and **B** Contrast computed tomography demonstrated a traumatic splenic artery pseudoaneurysm (**A** pre-contrast image; **B** arterial phase maximum intensity projection) **C** and **D**) Catheter angiography via the splenic artery (**C**) and segmental artery (**D**) demonstrate pseudoaneurysm (red circle and red arrow). **E** Post-embolization angiogram suggested successful occlusion of the culprit artery. Yellow arrow: Obsidio embolic. **F** Follow-up computed tomography demonstrated splenic infarct in the embolization territory (blue arrow) of the embolized territory. Yellow arrow: Obsidio embolic

A 48-year-old male presented with traumatic splenic artery pseudoaneurysm on CTA following motor-vehicle collision (Fig. 3). Catheter angiography revealed a 1.0 cm intraparenchymal pseudoaneurysm arising from a segmental branch of the superior aspect of the spleen. The culprit vessel was successfully embolized with 0.2 ml OCE via a microcatheter co-axial system. No procedural-related major complication occurred [7]. Postoperative CT demonstrated resolution of pseudoaneurysm and wedge-shaped infarct of the treated angiosome.

##  Discussion

This technical note demonstrated the effectiveness of OCE during selective embolization of acute intra-abdominal hemorrhage without complications. Its radiopacity allows real-time visualization under fluoroscopy and targeted delivery. Less than one vial of embolic (0.2-0.4 ml) was adequate to occlude the culprit vessel in all cases.

OCE is composed of nanocomposite hydrogel containing silicate nanoplatelets, a shear-thinning biomaterial that behaves like fluid with low viscosity under high pressure within the microcatheter during injection. Upon endoluminal delivery, it forms a bioactive scaffold within blood vessels via hydrogen bonding within the nanoparticles to strengthen the embolic structure. The subsequent vessel occlusion is achieved by the formation of a three-dimensional hierarchical gel network due to hydrophobic interactions above phase transition temperature [5]. In practice, slow and continuous injection allows distal penetration of the embolic due to its shear-thinning properties, whereas pulsed and forceful injection result in a plug-like deployment achieving instant proximal occlusion.

Compared to metallic coils that rely on the patient’s coagulation status, the vascular occlusion in OCE does not rely on intrinsic thrombosis for occlusion. In vitro studies on femoral arteries of have shown that OCE forms a complete, impenetrable cast of the vessel on histology, suggesting its suitability for patients on anticoagulation therapy or those with coagulopathy [4, 6]. Further, in cases where the target vessel is large and multiple coils are required, OCE can potentially occlude a longer segment of vasculature with less than one vial and obviates loading of additional embolic, which can be more time efficient and perhaps more cost effective compared to using multiple detachable coils. Typically, only a small proportion of one vial ranging from 0.1 to 0.3 ml was sufficient to achieve single vessel occlusion.

Although N-butyl cyanoacrylate glues, another commonly used embolic agent, also achieve occlusion independent of blood coagulability, the delivery of NBCA can be complicated by its highly adhesive property and very rapid polymerization [2, 8]. Catheter trapping may result in tissue injury and non-target embolization [9]. Compared to bulk polymers such as NBCA, OCE’s ability to adhere to the vessel wall is minimal, which has a higher procedural safety profile [4]. Catheter adherence did not occur in the present study. Whereas premature polymerization in NBCA may result in microcatheter occlusion, flushing with saline can effectively clear out OCE from catheters, which can be reused for embolization of additional vessels during the same treatment session.

Another major difference between OCE and NBCA or ethylene vinyl alcohol (EVOH)-based liquid embolics is the preparation and delivery system. While NBCA requires mixing with ethiodized oil and tantalum powder [10], the OCE comes pre-mixed in a sterile syringe ready for use. Compared to EVOH-based liquid embolics which require mixing and pre-loading with dimethylsulfoxide (DMSO), the OCE can be injected directly through standard angiographic catheters with no known risk of polymerization within or degradation of the catheter. Of note, the OCE is currently available in only a single formulation and viscosity is variable based on injection pressure; there is no recommendation on dilution of OCE.

The present report should be interpreted with several caveats. The sample size is small with limited follow-up length, precluding evaluation of its long-term safety and effectiveness. One concern regarding liquid embolic was distal ischemia. While upper gastrointestinal hemorrhage can be treated with OCE given the abundance of collateral supply, risk of tissue ischemia can be higher in embolization of lower gastrointestinal hemorrhage where OCE should be used with increased caution. While OCE is radio-opaque and the density of tantalum is low enough to reduce streak artifacts during CT follow-up examinations, its lesser radiopacity on fluoroscopy compared to metallic coils theoretically increase the risk of nontarget embolization. The tactile feedback during use of OCE is also significantly different from NBCA or EVOH-based embolics which may also increase the risk of nontarget embolization, particularly for operators who are unfamiliar with the relatively high resistance encountered during delivery. Further, despite its convenience, the cost of OCE was higher than gelfoam sponge and most pushable coils. In the authors’ opinion, the ideal scenario of using such OCE would be in the setting of coagulopathy, which warrants future research.

## Conclusions

In conclusion, OCE can be safely and effectively used for selective transarterial embolization. Its ease of use, ability to function independently of intact coagulation, and resistance to catheter entrapment render it a promising embolic agent. Future large cohort studies are warranted for further characterization of its safety and efficacy in different scenarios.

## Data Availability

Upon request.
